# Impact of prophylactic administration of Levosimendan on short-term and long-term outcome in high-risk patients with severely reduced left-ventricular ejection fraction undergoing cardiac surgery – a retrospective analysis

**DOI:** 10.1186/s13019-016-0556-2

**Published:** 2016-12-01

**Authors:** Philippe Grieshaber, Stella Lipp, Andreas Arnold, Gerold Görlach, Matthias Wollbrück, Peter Roth, Bernd Niemann, Jochen Wilhelm, Andreas Böning

**Affiliations:** 1 Department of Adult and Pediatric Cardiovascular Surgery, University Hospital Giessen, Rudolf-Buchheim-Str. 7, DE-35392 Giessen, Germany; 2 Department of Neurology, University Hospital Giessen, Giessen, Germany; 3 Department of Anaesthesiology and Intensive Care Medicine, University Hospital Giessen, Giessen, Germany; 4 Department of Internal Medicine, German Center for Lung Research, Justus Liebig University, Giessen, Germany

**Keywords:** Levosimendan, Cardiac surgery, Low cardiac output syndrome, High-risk patients

## Abstract

**Background:**

Patients with severely reduced left-ventricular ejection fraction carry a high risk of morbidity and mortality after cardiac surgery. Levosimendan can be used prophylactically in these patients having shown positive effects on short-term outcome. However, effects on long-term outcome and patient subgroups benefiting the most are unknown. We aim to address these topics with real-life data from our clinical practice.

**Methods:**

Two hundred eigthy eight patients with preoperative LVEF ≤ 35% underwent cardiac surgery with cardiopulmonary bypass between 2009 and 2013. Thereof, 246 were included in the matched analysis. Eigthy two patients received 12.5mg Levosimendan starting at induction of anesthesia. Outcomes of patients undergoing coronary artery bypass grafting surgery (*n* = 103), isolated valve surgery/ascending aortic surgery (*n* = 45) and those undergoing combination procedures (*n* = 98) were analyzed separately. Additionally, multivariate regression analysis was conducted in order to identify predictors of short-term outcome parameters for different subgroups of patients.

**Results:**

Thirty days mortality rates of 16% in the Levosimendan group and 21% in the control group (OR 0.7; 95%–CI 0.36–1.5; *p* = 0.37) were observed. Levosimendan showed a positive effect on postoperative renal function. A higher rate of new-onset atrial fibrillation (OR 4.0; 95%–CI 2.2–7-2; *p* < 0.0001) was observed in the Levosimendan group. Follow-up until three years postoperatively showed no differences in long-term survival between the groups.

**Conclusion:**

Prophylactic administration of Levosimendan did not affect overall short- and long-term outcomes. The value of prophylactic use of Levosimendan remains questionable and more data is needed to confirm subgroups that might benefit most.

**Electronic supplementary material:**

The online version of this article (doi:10.1186/s13019-016-0556-2) contains supplementary material, which is available to authorized users.

## Background

Levosimendan (LS) improves myocardial contractility without increasing myocardial oxygen demand by increasing calcium-sensitivity of the myocardial contractile units through binding to troponin C [1]. Furthermore, it induces systemic vascular and coronary artery dilation through activation of adenosine triphosphate (ATP)-dependent potassium channels in the vascular smooth muscle cells [2]. The effect of LS and its metabolite OR-1896 are reported to last up to seven days [1]. LS effects have been thoroughly investigated in the treatment for acutely decompensated chronic heart failure, showing positive results when compared to either dobutamine (RUSSLAN study) or placebo (LIDO study) [3, 4]. However, the REVIVE I and II studies showed adverse effects on those patients treated with LS [5].

Patients with preoperatively severely reduced ventricular contractility undergoing cardiac surgery with cardiopulmonary bypass (CPB) carry a substantial risk of postoperative low cardiac output syndrome with its consequences (organ malperfusion, shock, multi-organ failure). The advantageous properties of LS make it a promising therapeutic or even prophylactic option for prevention of these complications.

A number of small-sized prospective randomized trials have shown positive effects of prophylactic LS administration on postoperative cardiac performance [6, 7], renal function [8, 9], inflammation [10], demand on other inotropic drugs [11] as well as on short-term survival [12, 13]. However, the transferability of these excellent results to real-life practice has been questioned and, despite LS being one of the best-investigated drugs in cardiovascular medicine in the recent years, its prophylactic use in cardiac surgery has not become a widely established therapeutic concept. Furthermore, the potential durability of the LS effect resulting in improved long-term survival has not been shown so far [14, 15]. In order to give an update from the clinical routine and to generate hypotheses for further studies, we investigated, if the effect of prophylactic LS administration on short-term outcome can be confirmed in patients with preoperative LVEF ≤35% undergoing cardiac surgery outside the controlled setting of prospective trials. Furthermore we aimed to describe for the first time the effect of prophylactic LS on long-term survival in these patients. Also, dependence of the LS effect on complexity of the surgical procedure was investigated.

## Methods

### Study design

The present study was a retrospective single-center study. It aimed to describe the effect of prophylactic LS administration in patients with preoperative LVEF ≤35% undergoing cardiac surgery with the use of CPB.

### Ethics

The ethical committee of the Faculty of Medicine at Justus Liebig University Giessen, Germany approved the study. The trial was designed and conducted in accordance to the Declaration of Helsinki.

### Study population

**Fig. 1:**
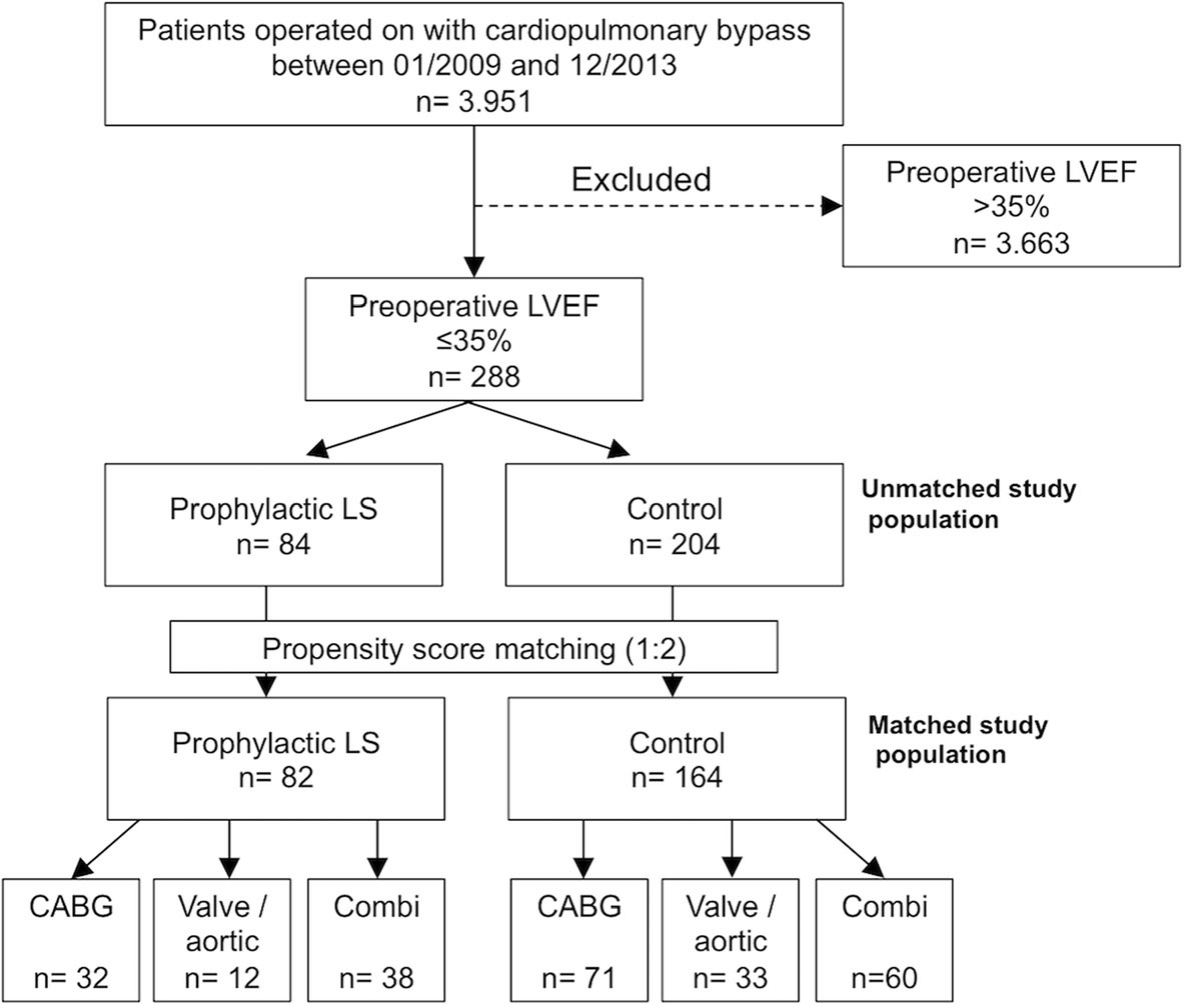
Patient inclusion flow chart. From 3.951 patients operated on in the inclusion period, 288 presented with preoperative LVEF ≤35%. Of these, 84 patients received prophylactic LS and 204 did not. After propensity score matching, 246 patients remained in the analysis with 82 patients in the LS group and 164 patients in the control group. Abbreviations: CABG: Coronary artery bypass grafting surgery, LS: Levosimendan, LVEF: Left-ventricular ejection fraction

Patients with preoperative LVEF ≤ 35% who underwent cardiac surgical procedures with CPB at our institution between 01/2009 and 12/2013 where identified from institutional patient records and data issued to the nationwide quality assurance program. Clinical records of these patients were reviewed and long-term survival was determined by obtaining the patients’ excerpts from the German federal residents’ registry. In order to correct for relevant differences in baseline characteristics, a 1:2-propensity score matching of LS group and control group was conducted (Fig. 1).

### Administration of LS

Patients received 12.5 mg LS via continuous intravenous infusion over 24 h starting at the induction of anesthesia. We did not apply an initial bolus. The treating surgeon and the anesthesiologist decided whether to administer prophylactic LS on an individual basis. Besides the LVEF, determined using the biplane Simpson method, criteria for LS use included preoperative state of cardiac compensation (clinical features evaluated during the preoperative visit: presence of dyspnea at rest, orthopnea, edema), hemodynamic reaction to induction of anesthesia (decrease of systolic blood pressure by >30mmHg without immediate stabilization after administration of inotropes, vasopressors or volume resuscitation) as well as complexity and estimated duration of the surgical intervention.

### Perioperative management

Perioperatively, medical circulatory support and hemodynamic monitoring was managed at the discretion of the treating intensive care physicians according to the relevant guidelines [16].

### Endpoints

Outcomes were compared between patients who received prophylactic LS and patients who did not. In order to clarify if the effect of prophylactic LS might be different depending on the complexity of surgery, outcomes were further analyzed separately for patients who underwent isolated coronary artery bypass grafting surgery (CABG group), isolated valve or aortic surgery (valve group) or combination procedures (combi group) respectively.

The primary endpoint of the trial was postoperative 30-days survival. Long-term survival functions were determined and compared using Kaplan-Meier estimation. Secondary endpoints included postoperative need for medical and mechanical circulatory support, renal function, new-onset atrial fibrillation as well as lengths of intensive care unit (ICU) stay and hospital admission.

### Statistics

In this retrospective study, a descriptive statistical analysis was performed using SPSS Version 22 (IBM, Armonk, USA), GraphPad Prism version 6 software (GraphPad Software, Inc., La Jolla, CA, USA) and R version 3.1.2. Patient characteristics were compared using Fisher’s exact test or Student’s *t*-test as appropriate. In order to correct for potential confounding baseline parameters between the LS group and the control group, propensity score matching of the groups was performed. Covariates included in the matching were age, gender, BMI, preoperative LVEF, pulmonary hypertension (categorized into no, moderate, severe), EuroSCORE II, preoperative chronic kidney injury (categorized into no, stadium 1, stadium 2, stadium 3 and stadium 4) and weight of the intervention (categorized in isolated CABG, isolated aortic/valve surgery, combination procedure). Hereafter, nearest neighbor matching in a 1:2 (LS : Control) fashion was performed. The maximum caliper between matched participants was set at 0.2. Group comparison for postoperative outcome parameters was performed by Fisher’s exact test or Student’s *t*-test between LS group and control group. Long-term survival functions were determined using Kaplan-Meier estimation and compared using the log rank test.

The effect of LS administration on the clinical outcome parameters ‘30-days survival’, ‘postoperative acute kidney injury (as defined by the AKIN-Criteria [17])’ and ‘postoperative new-onset atrial fibrillation within 24h post-surgery’ was additionally estimated by multivariate regression using generalized linear models. Survival data was fitted using Cox-proportional hazard models. Some metric predictors were log-transformed to linearize their relationship with the response (see Additional file 1 for detailed model formulations and coefficient tables).

## Results

### Study population

Of all patients who underwent cardiac surgical procedures with CPB at our institution between 01/2009 and 12/2013, 288 patients presented with preoperative LVEF < 35%. Thereof, 84 patients received LS prophylactically, 204 did not receive LS. Before propensity score matching, differences in preoperative renal function, previously known pulmonary hypertension and prevalence of recent acute myocardial infarction between the groups were observed. In the matched study population (*n* = 246; LS: *n* = 82; Control *n* = 164), these differences were eliminated and patients’ baseline characteristics were balanced between the groups (Table 1; Additional file 2). The procedural profiles showed a tendency towards more complex procedures in the LS group compared to the control group with 39% vs. 43% isolated CABG procedures and 46% vs. 37% combination procedures (*p* = 0.16). Isolated valve surgery and surgery on the thoracic aorta were evenly distributed between the groups (Table 1). Altogether, patients in both groups represented a high-risk patient population with EuroSCORE II values of 19.0% in the LS group and 17.1% in the control group ( *p* = 0.41) (details of the EuroSCORE II – relevant parameters: Additional file 2). Of note, CABG patients in the LS group had higher EuroSCORE II compared to the control group (15.8% vs.12.6%; *p* = 0.56) and valve group patients who received LS had lower EuroSCORE II values compared to the control group (13.7% vs. 20.9%; *p* = 0.37). Blood pressure at induction of anesthesia as well as duration of CPB and cardioplegic arrest were similar in both groups (Table 1).

**Table 1:** Baseline characteristics

		Unmatched study population			Matched study population		
Parameter	All patients *n* = 288 (100%)	LS + *n* = 84 (29.17%)	LS - *n* = 204 (70.83%)	*p*-value	LS + *n* = 82 (33%)	LS - *n* = 164 (67%)	*p*-value
Age [years]|; mean ± SD	69 ± 11	68 ± 11	70 ± 11	0.16	67 ± 11	68 ± 10	0.62
Sex [females] n (%)	70 (24)	19 (23)	51 (25)	0.76	19 (23)	41 (25)	0.88
Body mass index [kg/m^2^] mean ± SD	27 ± 7.0	27 ± 5.6	27 ± 7.5	0.71	27 ± 5.7	27 ± 8.2	0.81
Preoperative LVEF [%];mean ± SD	26 ± 7.3	26 ± 7.0	25 ± 8.0	0.14	26 ± 10	27 ± 11	0.47
Preoperative renal impairment; n (%) • Stage 2 (GFR 85-120ml/min.) • Stage 3 (GFR 51-85ml/min.) • Stage 4 (GFR <51ml/min.) • Stage 5 (Dialysis)	168 (58) 84 (29) 54 (32) 18 (11) 11 (6.5)	49 (58) 13 (27) 20 (41) 10 (20) 6 (12)	119 (58) 71 (60) 34 (29) 8 (6.8) 5 (4.2)	0.013	47 (57) 13 (28) 20 (43) 9 (19) 5 (11)	103 (63) 59 (57) 31 (30) 8 (7.8) 5 (4.9)	0.16
Preoperative atrial fibrillation; n (%)	96 (33)	29 (34)	67 (32)	0.48	29 (38)	57 (35)	0.67
Preoperative cardiopulmonary resuscitation; n (%)	14 (4.9)	4 (4.8)	10 (4.9)	1.00	4 (4.9)	9 (5.5)	1.00
Preoperative invasive ventilation; n (%)	22 (7.6)	5 (6.0)	17 (8.3)	0.67	5 (6.1)	15 (9.2)	0.49
Preoperative use of inotropes and vasoactive drugs; n (%) • Epinephrine • Norepinephrine • Milrinone • Dobutamine	48 (17) 12 (4.2) 18 (6.3) 4 (1.4) 14 (4.9)	9 (11) 1 (1.2) 3 (3.6) 1 (1.2) 4 (4.8)	39 (19) 11 (5.4) 15 (7.4) 3 (1.5) 10 (4.9)	0.11 0.41 1.00 1.00 0.42	9 (11) 1 (1.3) 3 (3.7) 1 (1.2) 4 (4.9)	23 (20) 9 (5.5) 14 (8.5) 2 (1.2) 8 (4.9)	0.12 0.10 0.19 1.00 1.00
EuroSCORE II [%]; mean ± SD • Isolated CABG group • Valve/aortic surgery group • Combi group	17.3 ± 16.4 14.3 ± 13.4 17.9 ± 19.8 21.4 ± 17.4	19.2 ± 16.4 17.8 ± 17.7 11.7 ± 10.2 23.0 ± 16.2	16.5 ± 16.4 12.9 ± 11.7 20.1 ± 21.8 20.4 ± 18.2	0.21 0.071 0.19 0.48	19.0 ± 16.6 15.8 ± 12.1 13.7 ± 10.0 20.1 ± 13.4	17.1 ± 17.3 12.6 ± 11.3 20.9 ± 22.8 21.4 ± 18.9	0.41 0.56 0.37 0.73
Type of Surgery; n (%) • Isolated CABG • Valve Surgery or Surgery on Thoracic aorta • Combination procedures	138 (48) 46 (16) 99 (34)	32 (38) 13 (15) 39 (46)	106 (52) 38 (19) 60 (29)	0.038 0.72 <0.01	32 (39) 12 (15) 38 (46)	71 (43) 33 (20) 60 (37)	0.76 0.16 0.19
Systolic blood pressure at induction of anaesthesia [mmHg]; median (IQR)	113 (101–128)	109 (98–127)	115 (100–130)	0.87	115 (101–123)	119 (103–126)	0.74
Diastolic blood pressure at induction of anaesthesia [mmHg]; median (IQR)	69 (60–82)	67 (59–81)	70 (59–83)	0.87	71 (61–87)	71 (65–79)	0.85
Extracorporeal circulation time [min.]; median (IQR)	98 (79–108)	93 (71–98)	103 (81–112)	0.24	114 ± 34	111 ± 26	0.58
Cardioplegic arrest time [min.]; median (IQR)	67 (51–89)	67 (50–90)	67 (53–86)	0.74	73 ± 30	72 ± 24	0.82

Demographics and intraoperative data of the study population before (left) and after propensity score matching (right). The unmatched population shows significant differences between LS and control group with more severe chronic renal impairment and a more complex procedural profile in the LS group. After matching, the groups are well balanced.

*Abbreviations*: *CABG* coronary artery bypass grafting surgery, *GFR* Glomerular filtration rate, *LS* Levosimendan, *LVEF* Left-ventricular ejection fraction

### Postoperative medical and mechanical circulatory support

The proportions of patients requiring postoperative medical support with epinephrine, norepinephrine, milrinone and dobutamine as well as the duration of application of these agents in these patients is shown in Table 2. Overall, 83% of LS patients compared with 68% of the control group patients required epinephrine (*p* < 0.001). Duration of norepinephrine support was longer in the LS group compared to the control group (median: 35h vs. 20h; *p* = 0.005). Otherwise, prevalence and duration of additional medical circulatory support did not differ between the groups.

**Table 2:** In-hospital outcomes

	Matched study population		
Parameter	LS + *n* = 82	LS - *n* = 164	*p*-value
Medical circulatory support			
• Epinephrine required; n (%)	68 (83)	112 (68)	<0.001
• Duration Epinephrine [h]; median (IQR)	19 (4.5–65)	10 (0–38)	0.01
• Nordrenaline required; n (%)	79 (96)	149 (91)	0.058
• Duration Nordrenaline [h]; median (IQR)	35 (15–68)	20 (11–47)	0.005
• Dobutamine required; n (%)	41 (50)	85 (52)	0.68
• Duration Dobutamine [h]; median (IQR)	25 (9–64)	12 (8–36)	0.83
• Milrinone required; n (%)	10 (12)	23 (14)	0.73
• Duration Milrinone [h]; median (IQR)	0.5 (0–14)	2.3 (0–18)	0.328
Mechanical circulatory support			
IABP insertion; n (%)			
• Intraoperatively	9 (11)	8 (4.9)	0.072
• Postoperatively	8 (9.8)	2 (1.2)	0.003
Duration IABP support [h]; median (IQR)	51 (40–73)	57 (26–75)	0.30
ECLS implantation; n (%)	6 (7.3)	6 (3.7)	0.40
Renal function			
Postoperative GFR [ml/min.]; mean (95%-CI)	68 (50–86)	70 (60–81)	0.48
Postoperative GFR/preoperative GFR [ml/min.]; mean (95%-CI)	1.0 (0.9–1.1)	1.0 (0.9–1.1)	0.47
Acute kidney injury; n (%)			
• AKIN I	14 (17)	37 (23)	0.22
• AKIN II	0	6 (3.7)	
• AKIN III	6 (7.3)	6 (3.7)	
New onset chronic dialysis; n (%)	1 (1.2)	3 (1.8)	1.00
Cardiac Injury			
AF within 24h post-OP; n (%)	62 (76)	72 (44)	<0.0001
CK until POD4 [U/l]; mean (95%-CI)	712 (368–1056)	619 (400–839)	0.402
CKMB until POD4 [U/l]; mean (95%-CI)	47 (23–72)	41 (29–54)	0.17
Length of stay			
Postoperative LOS ICU [h]; median (IQR)	150 (71–200)	139 (67–168)	0.047
Postoperative LOS total [d]; median (IQR)	10 (8.0–13)	11 (7.0–14)	0.94

Comparison between patients who received prophylactic LS and patients who did not. Patients in the LS group show increased need for medical and mechanical circulatory support (IABP) as well as an increased rate of postoperative atrial fibrillation, resulting in a prolonged demand for intensive care

*Abbreviations*: *AF* Atrial fibrillation, *AKIN* Acute kidney injury network, *CK* Creatinkinase, *CKMB* Creatinkinase, isoform MB, *CRP* C-reactive protein, *ECLS* Extracorporeal life support, *IABP* Intra-aortic balloon pump, *LOS* Length of stay, *LS* Levosimendan, *POD* post-operative day, *WBC* White blood cell count

Additional mechanical circulatory provided by intraoperative or postoperative initiation of intra-aortic balloon pump (IABP) was more frequently applied in the LS-group (21%) compared to the control group (6.1%; *p* = 0.0015). Postoperative ECLS (extracorporeal life support) implantation was necessary more often in the LS-group (6/82; 7.3%) compared to the control group without statistical significance (6/164; 3.7%; *p* = 0.40).

### Renal function

In the matched study population, preoperative renal function shows no significant differences between LS group and control group. However, a tendency towards more severely impaired renal function in the LS group is present (Table 1). In the postoperative course, glomerular filtration rates (GFR) showed an initial postoperative decline in both groups with a recovery within four days postoperatively (data not shown). Mean postoperative GFR and the ratio of postoperative GFR to preoperative GFR did not differ between LS-group and control group patients (Table 2). Multivariate regression analysis revealed a reductive effect of LS on the incidence of acute kidney injury (coefficient -1.37; *p* < 0.001). Interestingly, also preoperative IABP implantation had a comparable significant renoprotective effect (coefficient -1.69; *p* = 0.013) while aortic cross clamping time had an incremental effect on acute kidney injury (coefficient 1.45; *p* = 0.024) (Table 4).

### Postoperative arrhythmias

We observed an overall increased incidence of atrial fibrillation within 24h postoperatively in patients receiving prophylactic LS (62/82; 76%) compared to control group patients (72/164; 44%; *p* < 0.0001; OR 4.0; 95%-CI 2.2–7-2) (Table 2). This effect persisted in the differentiated comparison for CABG patients (OR 6.5; 95%-CI 1.8–21) and combination procedure patients (OR 2.5; 95%-CI 1.0–3.8). Linear regression confirmed prophylactic LS to be a predictor of postoperative AF (coefficient 0.86; *p* = 0.038). Also preoperative IABP insertion and aortic cross clamping time increased the risk of postoperative AF (Table 3).

**Table 3:** Linear modeling for preoperative predictors influencing survival 30 days postoperatively, postoperative atrial fibrillation and postoperative acute kidney injury

Responses	30-days survival			Postoperative AF within 24h			Postoperative acute kidney injury		
Parameter	Coefficient	Standard error	*p*-Value	Coefficient	Standard error	*p*-Value	Coefficient	Standard error	*p*-Value
Prophylactic LS	0.99	0.64	0.12	0.86	0.42	0.038	-1.37	0.53	0.0094
Preoperative IABP	0.40	0.69	0.56	2.19	0.70	0.0017	-1.69	0.68	0.013
Intraoperative IABP	-0.58	0.89	0.51	0.91	0.76	0.23	0.29	0.77	0.71
Postoperative IABP	-2.36	1.03	0.022	0.12	1.04	0.91	1.83	1.03	0.08
Aortic cross clamp time (log)	-1.04	0.84	0.21	1.40	0.57	0.014	1.45	0.64	0.024
EuroSCORE II (log)	-1.12	0.35	0.0012	-0.06	0.21	0.77	0.05	0.21	0.82
BMI (log)	-2.87	1.71	0.093	1.02	1.46	0.48	1.69	1.52	0.26
Preoperative admission to ICU	-0.34	0.66	0.60	0.42	0.59	0.48	0.95	0.59	0.11
Preoperative TNI	-0.09	0.68	0.90	-1.87	0.70	0.0074	-0.39	0.60	0.52
Preoperative CRP (log)	0.04	0.16	0.79	0.03	0.12	0.80	0.16	0.13	0.20
Preoperative white blood cell count (log)	-0.20	0.82	0.81	0.43	0.61	0.48	0.62	0.70	0.38

Multivariate regression analysis using generalized linear models was performed for the responses’30-days survival’, ‘postoperative new onset AF’ and ‘postoperative acute kidney injury’. LS has no significant effect on 30-days survival but contributes significantly to postoperative AF and significantly reduces postoperative acute kidney injury

*Abbreviations*: *AF*: Atrial fibrillation, *BMI* Body-mass index, *CRP* C-reactive protein, *IABP* Intra-aortic balloon pump, *ICU* Intensive care unit, *LS* Levosimendan, *TNI* Troponin-I

### In-hospital outcome

Patients in the LS group needed longer postoperative intensive care (median 150h; IQR 71-200h) compared to control group patients (median 139h; IQR 67-168h; *p* = 0.047) (Table 2). Postoperative length of hospital admission was 11 days (IQR 7–13 days), with no difference between LS group and control group.

### 30-days survival

Overall 30-days survival was reduced due to the patients’ high preoperative risk profile and a trend towards a higher survival rate in patients receiving prophylactic LS (69/82; 84%) compared to the control group (123/155; 79%; *p* = 0.40) was observed. Multivariate regression confirmed a tendency towards positive influence of LS on 30-days survival (coefficient 0.99; *p* = 0.12) while postoperative IABP implantation (coefficient -2.36; *p* = 0.022) and EuroSCORE II (coefficient -1.12; =0.0012) were significant predictors of reduced 30-days survival. All other investigated possible predictors did not influence 30-days survival (Table 3).

Different subgroup analyses for 30-days survival were conducted depending on (1) Procedural categories, (2) Stages of renal impairment, (3) Preoperative LVEF, (4) Categories of preoperative risk estimation using EuroSCORE II and (5) Concomitance of recent myocardial infarction. Here, tendencies towards pronounced beneficial effects of LS were observed for patients undergoing isolated valve/aortic surgery (30-days survival LS group: 92% vs. control group: 70%; *p* = 0.24), for patients with moderate chronic kidney injury/GFR 51–85ml/min. (30-days survival LS group: 85% vs. control group: 66%; *p* = 0.19), for patients with LVEF <25% (30-days survival LS group: 81% vs. control group: 71%; *p* = 0.34), patients who had no recent myocardial infarction (30-days survival LS group: 91% vs. control group: 83%; *p* = 0.22) and patients with EuroSCORE II > 23 (30-days survival LS group: 70% vs. control group: 51%; *p* = 0.19) (Table 4).

**Table 4:** 30-days survival

	30-days survival; n (%)		
Parameter	LS + *n* = 82	LS - *n* = 155	*p*-value
All patients *n* = 237	69/82 (84)	123/155 (79)	0.40
Subgroup: procedural categories			
*Isolated CABG*	26/32 (81)	58/68 (85)	0.77
*Isolated valve surgery / ascending aortic surgery*	11/12 (92)	23/33 (70)	0.24
*Combination procedures*	32/38 (84)	42/54 (78)	0.64
Subgroup analysis: renal impairment			
*GFR >120ml/min.*	32/35 (91)	50/59 (85)	0.53
*GFR 85–120ml/min.*	12/13 (92)	47/55 (86)	1.00
*GFR 51–85 ml/min.*	17/20 (85)	19/29 (66)	0.19
*GFR < 51ml/min.*	5/9 (56)	4/7 (57)	1.00
*Dialysis*	3/5 (60)	3/5 (60)	1.00
Subgroup analysis: preoperative LVEF			
*LVEF <25%*	29/36 (81)	46/65 (71)	0.34
*LVEF 25–30%*	21/23 (91)	41/48 (85)	0.60
*LVEF 31–35%*	19/23 (83)	36/42 (86)	0.72
Subgroup analysis: EuroSCORE II			
*EuroSCORE II <15*	37/39 (95)	84/91 (92)	0.72
*EuroSCORE II 15–17*	3/4 (75)	4/5 (80)	1.00
*EuroSCORE II 18–20*	2/2 (100)	4/5 (80)	1.00
*EuroSCORE II 21–23*	4/5 (80)	3/3 (100)	1.00
*EuroSCORE II >23*	16/23 (70)	20/39 (51)	0.19
Subgroup analysis: Recent myocardial infarction			
*No recent myocardial infarction*	50/55 (91)	72/87 (83)	0.22
*Recent myocardial infarction*	19/27 (70)	51/68 (75)	0.80

30-days survival data were available for 237/246 patients (96%). Overall, 30-days survival did not differ between patients who received prophylactic LS and patients who did not. In the subgroup analyses depending on procedural categories, renal impairment, preoperative LVEF, EuroSCORE II and recent myocardial infarction, no significant differences between LS group and control group were observed

*Abbreviations*: *CABG* Coronary artery bypass grafting surgery, *GFR* Glomerular filtration rate, *LS* Levosimendan, *LVEF* Left-ventricular ejection fraction

### Long-term survival

**Fig. 2:**
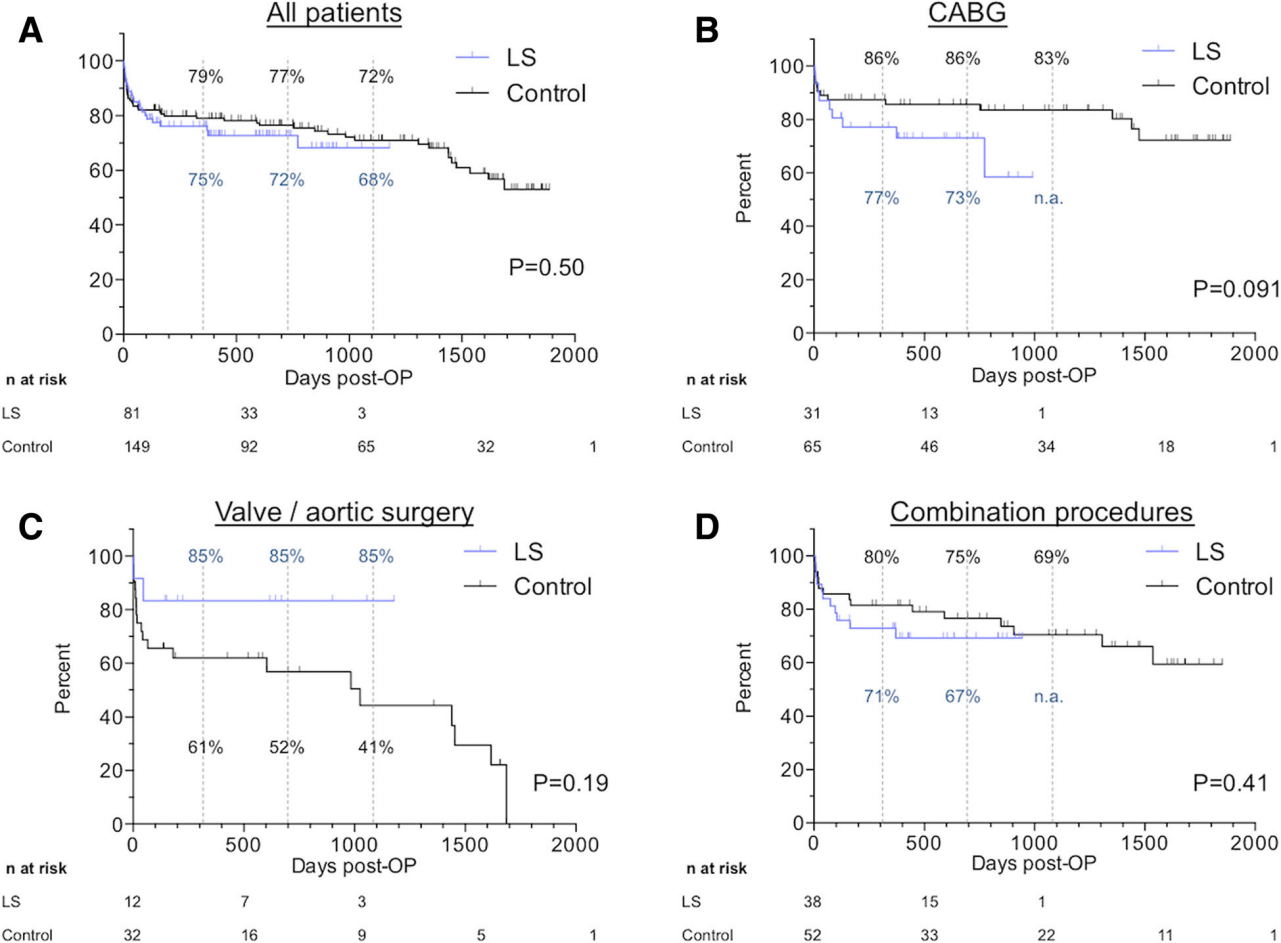
Kaplan-Meier estimation for long-term survival. Vertical lines at one, two and three years postoperatively. **a**. Overall survival. After an initial decline, survival remains stable after one two and three years in both groups. Survival curves do not differ. **b**. After isolated CABG, patients who received LS showed lower survival without statistical significance. **c**. After isolated valve or aortic surgery, LS patients show stable survival compared to reduced survival in the control group without statistical significance. D. After combination procedures, survival curves between the groups do not differ. Abbreviations: CABG: Coronary artery bypass grafting surgery, LS: Levosimendan

Median follow-up time was 610 days (IQR 130–1192 days). Follow up was complete for 238/246 (97%) patients. Kaplan-Meier estimation was (1) applied for all patients and (2) differentiated into the categories of surgical procedures (Fig. 2). Survival curves showed an initial decline in survival for both groups, representing the immediate postoperative period. Subsequently, a slower decline was observed in both groups. Overall survival was 76% (LS) and 79% (control) at one year, 73% (LS) and 76% (control) at two years and 68% (LS) and 71% (control) at three years postoperatively. In isolated CABG, LS patients tended to reduced long-term survival compared with the control group (Fig. 2b), contrarily to valve-group patients whose long-term survival exceeded the control group’s survival (Fig. 2c).

## Discussion

We present real-world data from a high-risk patient collective undergoing cardiac surgery with CPB. LS application had no significant effect on overall 30-days survival which is on one hand contradictory to previous studies showing positive effects of prophylactic LS on short-term survival [11, 12]. On the other hand, recent meta-analysis of the available data revealed conflicting results on whether or not LS reduces mortality [18, 19]. A differentiated analysis of our patient subgroups showed tendencies towards positive effects of LS on 30-days survival in patients undergoing valve/aortic or combination procedures, patients with moderate chronic renal impairment, patients who had no recent myocardial infarction, patients with LVEF <25% and patients with EuroSCORE II risk estimation scores >23. These findings suggest differential effects of LS depending on procedure-related factors as well as on patient-related factors. However, the size and design of our study disables any final conclusion upon the significance of these aspects.

LS reduced the incidence of postoperative acute renal failure significantly. This result is consistent with previous studies showing renoprotective properties of LS in cardiac surgical patients [8, 14].

Surprisingly, LS patients showed an excessively high rate of atrial fibrillation within 24h postoperatively compared to the control group (85% vs. 46%). As LS does not increase intracellular calcium levels, it has been postulated that LS might be advantageous compared to traditional inotropes concerning the pro-arrhythmic effects. However, our result is consistent with the SURVIVE I and II trials, which also showed higher rates of arrhythmias in ADHF-patients treated with LS [5].

Postoperative need for medical circulatory support was increased in the LS group, resulting in a prolonged need for intensive care. According to the lack of positive short-term-effects of LS, long-term survival up to three years postoperatively was not improved. Our observations are consistent with a previous study by Lahtinen et al. that compared prophylactic LS with placebo and reported similar survival in both groups 6 months postoperatively [20].

In summary, this study showed no or at least substantially weaker effects of prophylactic LS compared to previous reports. It is well arguable, how retrospective data should be weighed compared to methodically superior prospective data: Prospective randomized-controlled studies are the gold standard for evaluation of clinical effects of single interventions. However, this controlled setting differs substantially from the clinical routine setting. This might, among others, result in compliance bias and contamination bias with consecutively reduced external validity [21–24]. Thus, retrospective data, reflecting real-life practice, could give important additional information in order to classify the value of an intervention in the daily routine and to generate hypotheses for further studies.

Some aspects could be explanatory for a reduced overall LS-effect in the routine setting: First, the effect of LS might have been underutilized by our therapeutic regime. We did not apply an initial loading dose prior to continuous infusion over 24h: A current expert opinion paper states that an initial bolus at induction of anesthesia is a feasible option without emphasizing explicit positive effects of loading dose administration [25]. Contrarily, application of a LS bolus carries the risk of acute vasodilation and hemodynamic destabilization and has been shown to increase mortality in different clinical settings [14]. Therefore, LS bolus administration has not been practiced in our clinical routine. As we did not adjust LS dosing for renal impairment, overdosing and increased side effects might have resulted in some patients with severe renal impairment.

Second, the timing of LS administration appears to be critical: In our practice, LS was started after induction of anesthesia, when LVEF was determined using transoesophageal echocardiography. Without initial bolus, a steady-state is achieved after 4h [25]. As median operation time in our study population was 157 min., the full effect of LS might not have been reached at the critical time points, namely weaning from cardiopulmonary bypass and immediate postoperative phase.

Third, patient selection might have been too restrictive. We only administered prophylactic LS to patients with severely reduced LVEF. It might be argued that these patients’ precondition impedes positively influencing their postoperative outcome. However, we even observed a trend towards more pronounced survival benefit 30 days postoperatively in patients with preoperative LVEF < 25%. This observation is consistent with a meta-analysis of randomized controlled trials on prophylactic LS in cardiac surgery patients by Harrison et al. suggesting that patients with preoperatively severely reduced LVEF benefit in a greater extent from prophylactic LS compared to patients with preoperatively normal LVEF [26].

On the other hand, methodical limitations of this study could have biased the results:

First, this is a retrospective analysis with all its limitations. In most of the patients, no continuous cardiac-output monitoring was applied. Consecutively, medical circulatory support management was mainly based on individual decisions by the treating physicians. This might have contributed to suboptimal management of inotropes and vasopressors. The study included a total of 288 patients, a relatively small number of patients, with only 84 patients receiving prophylactic LS. This study population might have been too small to show possibly significant effects of prophylactic LS properly.

A major limitation of this study is, that the criteria for administration of LS were loose and decision individually taken by the treating surgeon and anesthesiologist during induction of anesthesia, which might have led to sampling bias with sicker patients in the LS group. However, after propensity score matching, risk estimation using EuroSCORE II and other baseline characteristics showed no relevant difference between the groups. Nevertheless, unknown confounders could still have biased the results.

## Conclusions

Prophylactic LS application in high-risk patients with preoperative LVEF ≤35% undergoing cardiac surgery had no relevant positive effect on short- and long- term survival. Although LS application was associated with improved postoperative renal function, the occurrence of postoperative atrial fibrillation was even increased compared to patients who did not receive any preoperative preconditioning. Optimal utilization of potential LS effects and translation of these effects into long-term benefit has not been achieved yet as critical questions are still unanswered: It remains unclear when and how to start prophylactic LS administration and which patients undergoing which procedures benefit most from this intervention. Furthermore, comparisons to established preconditioning concepts (e.g., prophylactic intra-aortic balloon counterpulsation) have to be substantiated in future studies. Based on the results of this study, a prospective trial with 462 patients per group would be needed to generate definitive results. Until then, reluctance to include prophylactic LS application, a cost-intensive (3.725€ per standard dose (12.5mg) [27]) non-subsidized intervention, into clinical routine seems justified.

## Additional files

Additional file 1: Generalized linear models Symbolic representation of the linear models used to estimate the effect of prophylactic Levosimendan and other predictors on 30-days survival, on postoperativ acute kidney injury (AKIN I-III) and on postoperative new-onset atrial fibrillation respectively. (DOCX 81 kb)
Additional file 2: EuroSCORE II-relevant baseline characteristics of the unmatched and matched study populations Before matching, LS group and control group significant differences with more frequent occurrence of ‘acute myocardial infarction’ in the control group and more severe pulmonary hypertension in the LS group. After matching, these significant differences were eliminated. (DOCX 73 kb)

## Abbreviations

CABG: Coronary artery bypass grafting surgery
CI: Confidence interval
CPB: Cardiopulmonary bypass
ECLS: Extracorporeal life support
GFR: Glomerular filtration rate
ICU: Intensive care unit
IQR: Interquartile range
LS: Levosimendan
LVEF: Left-ventricular ejection fraction
OR: Odds ratio
