# Supplementary material for: Impact of prophylactic administration of Levosimendan on short-term and long-term outcome in high-risk patients with severely reduced left-ventricular ejection fraction undergoing cardiac surgery – a retrospective analysis
Source: J Cardiothorac Surg. 2016 Dec 1;11:162. doi: 10.1186/s13019-016-0556-2 (PMC5131413; doi:10.1186/s13019-016-0556-2)
Supplement: Additional file 1: — Generalized linear models Symbolic representation of the linear models used to estimate the effect of prophylactic Levosimendan and other predictors on 30-days survival, on postoperativ acute kidney injury (AKIN I-III) and on postoperative new-onset atrial fibrillation respectively. (DOCX 81 kb) [file 13019_2016_556_MOESM1_ESM.docx]

**Supplement 1. Generalized linear models** Symbolic representation of the linear model**s** used to estimate the effect of prophylactic Levosimendan and other predictors on 30-days survival, on postoperativ acute kidney injury (AKIN I-III) and on postoperative new-onset atrial fibrillation respectively.

Abbreviations: IABP, intra-aortic balloon pump; ICU, intensive care unit; TNI, troponin I, CRP, C-reactive protein.

30-days survival:

*(30-days survival) ~ (Levosimendan administration) + (preoperative IABP) + (intraoperative IABP) + (postoperative IABP) + (recent myocardial infarction) + log(aortic cross clamping time) + log(EuroSCORE II) + log(Body mass index) + (preoperative admission to ICU) + (preoperative TNI>1µg/l) + log(preoperative CRP) + log(preoperative Leucocytes)*

Acute kidney injury:

*(Acute kidney injury (AKIN I+II+III)) ~ (Levosimendan administration) + (preoperative IABP) + (intraoperative IABP) + (postoperative IABP) + (recent myocardial infarction) + log(aortic cross clamping time) + log(EuroSCORE II) + log(Body mass index) + (preoperative admission to ICU) + (preoperative TNI>1µg/l) + log(preoperative CRP) + log(preoperative Leucocytes)*

Postoperative new-onset atrial fibrillation:

*(Acute kidney injury (AKIN I+II+III)) ~ (Levosimendan administration) + (preoperative IABP) + (intraoperative IABP) + (postoperative IABP) + (recent myocardial infarction) + log(aortic cross clamping time) + log(EuroSCORE II) + log(Body mass index) + (preoperative admission to ICU) + (preoperative TNI>1µg/l) + log(preoperative CRP) + log(preoperative Leucocytes)*
