# Supplementary material for: Impact of prophylactic administration of Levosimendan on short-term and long-term outcome in high-risk patients with severely reduced left-ventricular ejection fraction undergoing cardiac surgery – a retrospective analysis
Source: J Cardiothorac Surg. 2016 Dec 1;11:162. doi: 10.1186/s13019-016-0556-2 (PMC5131413; doi:10.1186/s13019-016-0556-2)
Supplement: Additional file 2: — EuroSCORE II-relevant baseline characteristics of the unmatched and matched study populations Before matching, LS group and control group significant differences with more frequent occurrence of ‘acute myocardial infarction’ in the control group and more severe pulmonary hypertension in the LS group. After matching, these significant differences were eliminated. (DOCX 73 kb) [file 13019_2016_556_MOESM2_ESM.docx]

**Supplement 2.** **EuroSCORE II-relevant baseline characteristics of the unmatched and matched study populations** Before matching, LS group and control group significant differences with more frequent occurrence of ‘acute myocardial infarction’ in the control group and more severe pulmonary hypertension in the LS group. After matching, these significant differences were eliminated.

Abbreviations: LS: Levosimendan; NYHA: New York Heart Association.

|  | **Unmatched study population** | | | **Matched study population** | | |
| --- | --- | --- | --- | --- | --- | --- |
| **Parameter** | **LS +**  n=84 (29.17%) | **LS -**  n=204 (70.83%) | **p-value** | **LS +**  n=82 (33%) | **LS -**  n=164 (67%) | **p-value** |
| **Extracardiac arteriopathy; n (%)** | 19 (23) | 62 (30) | 0.20 | 19 (23) | 51 (31) | 0.13 |
| **Poor mobility; n (%)** | 5 (5.9) | 23 (11) | 0.16 | 5 (6.1) | 19 (11.6) | 0.25 |
| **Previous cardiac surgery; n (%)** | 10 (12) | 20 (9.8) | 0.67 | 10 (12) | 16 (10) | 0.58 |
| **Chronic lung disease; n (%)** | 14 (17) | 44 (22) | 0.62 | 16 (20) | 34 (21) | 0.87 |
| **Active Endocarditis; n (%)** | 0 | 3 (1.5) | 0.56 | 0 | 3 (1.8) | 0.55 |
| **Critical preoperative state; n (%)** | 47 (56) | 75 (37) | 0.12 | 45 (55) | 80 (49) | 0.42 |
| **Recent myocardial infarction; n (%)** | 27 (32) | 97 (48) | **0.019** | 27 (32) | 71 (43) | 0.13 |
| **Diabetes mellitus; n (%)**   - **Without insulin treatment** - **With insulin treatment** | 31 (37)  16 (19)  15 (18) | 90 (44)  29 (24)  41 (20) | 0.55 | 31 (38)  16 (20)  15 (18) | 77 (47)  35 (21)  42 (17) | 0.38 |
| **Congestive heart failure; n (%)**   - **NYHA I** - **NYHA II** - **NYHA III** - **NYHA IV** | 0  5 (5.9)  42 (50)  29 (35) | 1 (0.5)  31 (15)  92 (45)  68 (33) | 0.15 | 0  5 (6.1)  42 (51)  27 (33) | 1 (0.6)  26 (16)  78 (48)  49 (30) | 0.17 |
| **CCS class IV angina; n (%)** | 42 (50) | 119 (58) | 0.24 | 42 (51) | 91 (55) | 0.59 |
| **Pulmonary hypertension; n (%)**   - **No** - **Moderate** - **Severe** | 77 (92)  3 (3.6)  4 (4.8) | 186 (91)  18 (8.8)  0 | **0.0025** | 76 (92)  3 (3.7)  3 (3.7) | 148 (91)  8 (4.7)  0 | 0.23 |
| **Urgency of operation; n (%)**   - **Elective** - **Urgent** - **Emergency** | 37 (44)  40 (48)  7 (8.3) | 97 (48  75 (36)  32 (16) | 0.18 | 37 (45)  38 (46)  7 (8.5) | 84 (51)  58 (35)  22 (13) | 0.20 |
